# Outcome of Two Corneal Collagen Crosslinking Methods in Bullous Keratopathy due to Fuchs' Endothelial Dystrophy

**DOI:** 10.1155/2014/463905

**Published:** 2014-11-17

**Authors:** Omur O. Ucakhan, Ayhan Saglik

**Affiliations:** Department of Ophthalmology, Ankara University Faculty of Medicine, Mamak Caddesi, Cebeci, 06340 Ankara, Turkey

## Abstract

Four eyes of 2 patients with corneal edema due to Fuchs' endothelial dystrophy were treated with CXL using the standard protocol. Since no improvement in visual acuity, corneal clarity, thickness, or pain sensation was evident in any eye at month 12, 2 eyes of the 2 patients were retreated, this time, following intraoperative corneal dehydration with glycerol 70% drops. This retreatment also failed to produce any significant effect on vision, corneal clarity, thickness, or pain in either eye. Collagen crosslinking with the current protocols may not be effective in the management of eyes with corneal edema due to Fuchs' endothelial dystrophy. Further studies are required to establish the efficacy of CXL and optimize the technique and/or dehydration method utilized in these cases.

## 1. Introduction

Collagen crosslinking (CXL) of the cornea by combined topical riboflavin and ultraviolet A (UVA) application is a widely accepted mode of treatment in keratoconus halting the progression of the disease. Few years after its first description, Wollensak et al. [1] noticed the dehydrating effect of CXL on porcine corneal tissue and suggested the reason to be the formation of additional intra- and interfibrillary chemical bonds within corneal collagen preventing further fluid influx and stromal swelling. Since then, a handful of studies evaluated the effect of CXL in bullous keratopathy [2–7]. Despite different methodologies, most studies showed a temporary improvement in corneal clarity and pain.

We herein report our experience with CXL using two different methods in 2 patients with Fuchs' endothelial dystrophy.

## 2. Case Reports

### 2.1. Case 1

**Figure 1:**
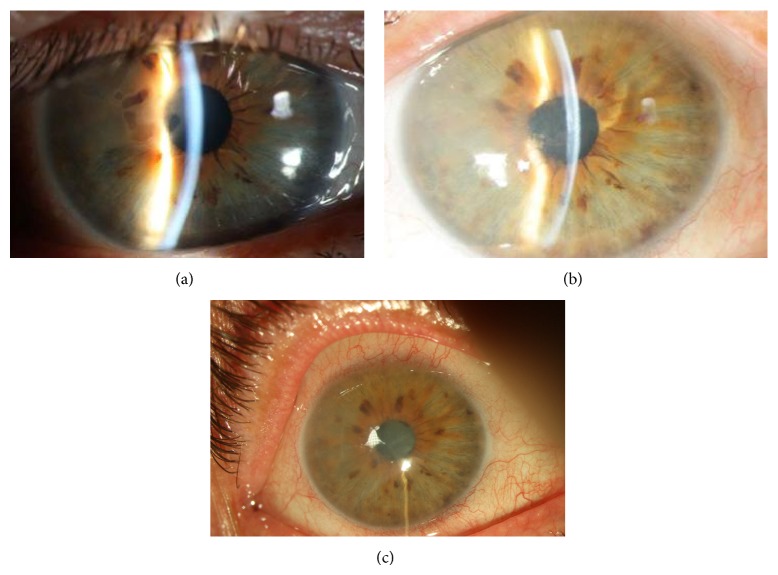
(a) Case 1: slit lamp biomicroscopy picture of the right eye before CXL. (b) Case 1: slit lamp biomicroscopy picture of the right eye 12 months after CXL. (c) Case 1: slit lamp biomicroscopy picture of the right eye 3 months after repeat CXL.

A 67-year-old man with the diagnosis of Fuchs' endothelial dystrophy presented with low visual acuity and pain in both eyes. He had been fit with bandage soft contact lenses for 12 months for painful bullous keratopathy. The visual acuities were 20/63 in both eyes and did not improve with refraction. Both corneas appeared thick with epithelial macrobullae and stromal subepithelial scarring. There was mild nuclear sclerosis in both eyes and dilated fundus examination of both eyes was normal (Figure 1(a)). The central corneal thicknesses (CCT) of the right and left eyes were 703 and 613 *μ*m (Micropac 200P, Sonomed Inc., USA) and the endothelial cell counts (ECC) were 815 ± 25 and 952 ± 32 cells/mm^2^ using laser-scanning confocal microscopy (Heidelberg Retina Tomograph II with Rostock Cornea Module, Germany).

Since the patient was tired of using bandage contact lenses to both eyes, CXL was offered to him as a possible alternate means of transient pain relief while awaiting for a corneal transplant procedure. Corneal collagen crosslinking using the standard protocol reported by Wollensak et al. [8] was performed on both corneas of this patient (365 nm, 3 mW/cm^2^) (UV-X, Germany) 1-week apart, with placement of bandage contact lenses and usage of topical antibiotics (starting at the day of surgery) and steroids (after reepithelization) postoperatively. Complete reepithelization occurred at day 7 in each eye at which time the contact lenses were removed; however, since the patient complained of pain, the contact lenses had to be replaced at postoperative week 2 in the left and postoperative week 3 in the right eye and continued to be used thereafter until month 12 with monthly replacements. At postoperative month 1, there was 1 Snellen line improvement of vision in each eye, and although at slit lamp biomicroscopy macrobullae were present in both eyes, the CCT decreased by about 100 *μ*m in the right and 50 *μ*m in the left eye (Figure 1(b)). The visual acuity and CCT deteriorated back to preoperative levels by postoperative month 3 in both eyes and remained stable until postoperative month 12. The ECC did not change through the follow-up period. A repeat CXL procedure was performed in the right eye of the patient at month 12. At that time, the visual acuity was 20/63, CCT was 722 *μ*m, and the ECC was 796 cells/mm^2^. At the time of surgery, following riboflavin 0.1% application every 2 minutes for 30 minutes, CCT was measured as 634 *μ*m. This time, in order to dehydrate the cornea, glycerol 70% drops were applied every 15 seconds until the corneal thickness was 450 *μ*m. This was followed by UVA irradiation (365 nm, 3 mW/cm^2^) for 30 minutes, and the CCT was monitored every 5 minutes to make sure the thickness never exceeded 450 *μ*m. At the end of the procedure, the CCT was measured as 421 *μ*m. Reepithelization was complete by day 6, and the bandage contact lens was removed; however, the patient returned 2 days later with the complaint of pain. Micro and macrobullae were seen at slit lamp biomicroscopy and the patient was again fit with a contact lens. The visual acuity was 20/63 throughout the postoperative follow-up (month 6); the CCT improved about 100 *μ*m at postoperative month 1 but deteriorated to 652 *μ*m, 710 *μ*m at months 3 and 6 (Figure 1(c)); and the ECC was remained stable until month 6. The patient did not wish to undergo a repeat CXL procedure in the left eye.

### 2.2. Case 2

**Figure 2:**
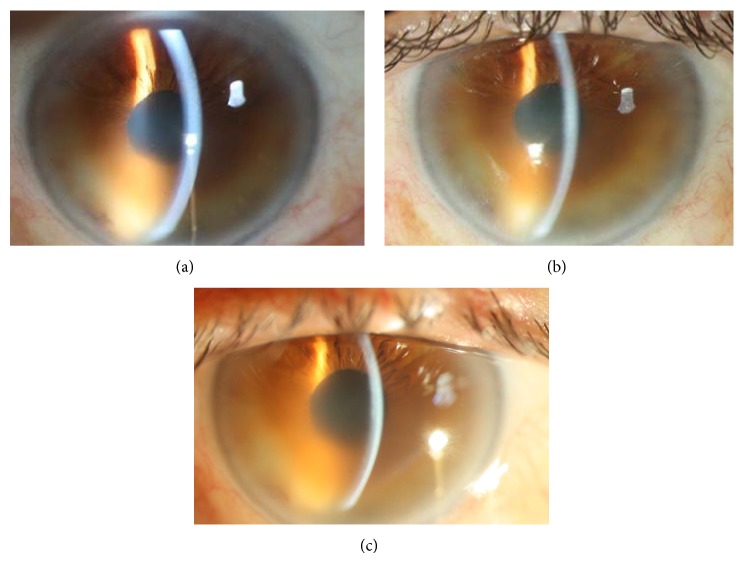
(a) Case 2: slit lamp biomicroscopy picture of the right eye before CXL. (b) Case 2: slit lamp biomicroscopy picture of the right eye 12 months after CXL. (c) Case 2: slit lamp biomicroscopy picture of the right eye 3 months after repeat CXL.

A 66-year-old man with the diagnosis of Fuchs' endothelial dystrophy presented with low visual acuity and pain in both eyes. The visual acuities were 20/63 in both eyes and did not improve with refraction. Both corneas appeared thick central and inferiorly with epithelial microbullae and stromal edema (Figure 2(a)). There was mild nuclear sclerosis in both eyes and dilated fundus examination of both eyes was normal. The central corneal thicknesses (CCT) of the right and left eyes were 575 and 550 *μ*m and the endothelial cell counts (ECC) were 1722 ± 32 and 1711 ± 36 cells/mm^2^.

Since the patient did not wish to be fit with bandage contact lenses for pain relief, he was offered CXL. Standard CXL was performed on both corneas of this patient 1-week apart. Reepithelization was complete by day 4 in each eye and the bandage contact lenses were removed. Postoperatively, the visual acuities in both eyes were 20/63 at all follow-up examinations from month 1 to month 12. The CCT measurements were 580, 586, 579, and 582 *μ*m in the right eye and 570, 580, 578, and 585 *μ*m in the left eye at months 1, 3, 6, and 12 (Figure 2(b)). The ECC was 1705 ± 43 and 1703 ± 40 cells/mm^2^ in the right and left eyes at postoperative month 12. Since no clinical improvement could be obtained, a repeat CXL procedure was performed in the left eye of this patient. During surgery, following riboflavin 0.1% application for 30 minutes, CCT was measured as 534 *μ*m. Glycerol 70% drops were applied every 15 seconds until the corneal thickness was 445 *μ*m. This was followed by UVA irradiation (365 nm, 3 mW/cm^2^) for 30 minutes. At the end of the procedure the CCT was measured as 421 *μ*m. Reepithelization was complete by day 4, and the contact lens was removed. The visual acuity was again 20/63 throughout the postoperative follow-up. There was no change in corneal edema at slit lamp biomicroscopy, no change in CCT, and no change in ECC until the last follow-up at month 6 (Figure 2(c)). The patient did not wish to undergo a repeat CXL procedure in the right eye.

Table 1 summarizes the results of the CXL procedures performed in Cases 1 and 2.

## 3. Discussion

In this study, initially CXL with the standard protocol was applied to 2 eyes of 2 patients with painful corneal edema due to Fuchs' endothelial dystrophy. Case 1 had initial reduction in CCT at month 1 in both eyes, followed by rapid deterioration afterwards, and no change in pain sensation at any timepoint. Case 2 had no change in vision or corneal clarity or thickness anytime following surgery. One eye of each patient was then treated with the modified protocol suggested by Hafezi et al. [7]. In Case 1, apart from a 100 *μ*m decrease in corneal thickness at month 1, there was no improvement in visual acuity, corneal edema, or pain sensation during 6 months of follow-up. Case 2 did not experience any improvement in either visual acuity, corneal clarity, or thickness anytime after repeat surgery. The ECCs did not change throughout the follow-up. To our knowledge, our cases are the only ones reported in the literature who did not benefit from CXL with any method even temporarily.

In initial studies, CXL using the standard protocol has been shown to have a transient beneficial effect on vision, corneal thickness, clarity, and pain scores [1, 4–6]. To improve the outcome of the procedure, Wollensak et al. [3] performed CXL following dehydration with glucose 40% eye drops (three times daily) one day prior to surgery in 3 eyes of 3 patients and reported improvement in bullous changes, pain sensation, and CCT at the end of 8 months. Hafezi et al. [7] tried another modification and dehydrated the cornea following epithelial debridement and 30-minute application of riboflavin 0.1% whenever the corneal thickness exceeded 450 *μ*m, with glycerol 70% solution applied every 5 seconds for 2 minutes. One eye of a patient with early Fuchs' dystrophy was treated and one-month after surgery the authors noted 2-line improvement in vision with about 100 *μ*m thinning in corneal thickness which was reported to be stable at month 3.

Arora et al. [9] reported their experience with CXL in pseudophakic bullous keratopathy (PBK) in 24 eyes. They noted that the mean visual acuity, symptoms, and pachymetry readings improved significantly at 1 month postoperatively in all patient eyes, whereas the symptoms and findings deteriorated thereafter with the effect being maintained in only 9 out of 12 patient eyes at month 3. The authors concluded that the effect of CXL decreases with time and depends on disease severity.

In a more recent study, Sharma et al. [10] reported their results in 50 eyes with PBK using CXL (standard protocol). The authors reported that pain scores decreased significantly on day 7 and started to regress towards month 6, and the mean CCT decreased significantly at postoperative month 1 and remained stable throughout the 6-month follow-up, whereas, visual acuity improved during the first 3 months and then started to deteriorate to preoperative levels at month 6. Corneal bullae recurred in 44% of eyes at the end of the follow-up.

The reason for ineffectivity of CXL with both the standard protocol and the protocol suggested by Hafezi and coauthors in our study may be due to differences in patient characteristics or surgical techniques. As suggested previously, efficacy of CXL in bullous keratopathy may tend to be less pronounced in advanced keratoconus [9, 10]. Our first patient was an advanced case. However, the second patient had mild to moderate edema. The technique used for CXL was not mentioned in Aurora's study, and although similar grossly, the CXL technique may show minor changes from one center to the other. Even with the same irradiation device, the exact distance of the device from the eye or the frequency of application of riboflavin 0.1% (every 2 or 3 or 5 minutes) may vary, and these minor differences may perhaps account for the different outcomes in such cases.

With CXL, the formation of covalent bonds between adjacent collagen fibrils leads to compaction of the corneal stroma, decreasing the potential space for fluid accumulation in the edematous cornea. This provides symptomatic relief and improvement in vision [1, 8]. However, CXL does not address the cause of corneal edema in Fuchs' dystrophy. On the other hand, reduced visual function in advanced bullous keratopathy is because of a reduction in stromal transparency as well as the irregular astigmatism created by the edematous epithelium. CXL might address stromal edema; yet, there is little evidence it addresses epithelial edema, since the epithelial edema of bullous keratopathy is not because of increased corneal stromal thickness per se. Furthermore, from experience with corneal ectasia, CXL is known to induce immunological reactions in certain eyes [11]. The significance of any immunological alteration of the cornea induced by CXL in regard to the survival of future corneal transplants remains unknown. Also, CXL may cause scarring of the cornea which may affect the success of future endothelial transplants. Further studies are required to establish the efficacy of CXL in corneal edema and to standardize the most efficient technique and/or dehydration method utilized in corneal edema. Until then, although CXL is a minimally invasive procedure, we believe risk-benefit analysis should be done with caution in eyes with corneal edema since these eyes are more prone to infection.

## Figures and Tables

**Table 1:** Summary of the outcomes of collagen crosslinking procedures in Case #1 and Case #2.

		Eye	Pre-CXL	Post-CXL Mo-1	Post-CXL Mo-12	Post-repeat CXL Mo-6
Visual acuity	Case #1	OD	20/63	20/50	20/63	20/63
		OS	20/63	20/50	20/63	—
	Case #2	OD	20/63	20/63	20/63	—
		OS	20/63	20/63	20/63	20/63

Pachymetry (*µ*m)	Case #1	OD	703	600	722	710
		OS	613	562	614	—
	Case #2	OD	575	580	582	—
		OS	550	570	585	586

Endothelial density (cells/mm^2^)	Case #1	OD	815	801	796	791
		OS	952	947	950	—
	Case #2	OD	1722	1719	1705	—
		OS	1711	1709	1701	1698

Pain relief	Case #1	OD	NA	Nil	Nil	Nil
		OS	NA	Nil	Nil	—
	Case #2	OD	NA	Nil	Nil	—
		OS	NA	Nil	Nil	Nil
